# Current Surge of Severe Acute Respiratory Syndrome Coronavirus-2 (SARS-CoV-2) Variants in China Could Be Threatening as the Asian Games 2023 Flags Off in September: Foolproof Cautionary Measures Are Suggested

**DOI:** 10.7759/cureus.45591

**Published:** 2023-09-20

**Authors:** Ranjan K Mohapatra, Ahmed Mahal, Snehasish Mishra, Gaganendu Dash, Lawrence S Tuglo, Venkataramana Kandi

**Affiliations:** 1 Chemistry, Government College of Engineering Keonjhar, Keonjhar, IND; 2 Medical Biochemical Analysis, College of Health Technology, Cihan University-Erbil, Erbil, IRQ; 3 School of Biotechnology, KIIT University, Bhubaneswar, IND; 4 Sports, KIIT University, Bhubaneswar, IND; 5 Nutrition and Dietetics, University of Health and Allied Sciences, Ho, GHA; 6 Clinical Microbiology, Prathima Institute of Medical Sciences, Karimnagar, IND

**Keywords:** viral transmission, international sports event, medical emergency, severe acute respiratory syndrome coronavirus-2 (sars-cov-2), coronavirus disease-19 (covid-19)

## Abstract

Despite normalcy having almost returned in the lives of people throughout the world post-coronavirus disease-19 (COVID-19) pandemic caused by the severe acute respiratory syndrome coronavirus-2 (SARS-CoV-2), the danger still looms over the fears of development and propagation of a newer SARS-CoV-2 variant. The movement of people globally has reached the pre-pandemic level, and this augmentation increased surveillance regarding the emergence of lethal SARS-CoV-2 variants. International sports events are among the potential avenues where the virus could cause serious impact. Therefore, the organization of such events should be planned and executed meticulously to avoid viral transmission and minimize the health effects of infections on the sportspersons and the local people. Additionally, there could be dissemination of the infections to the native countries of the participants and visitors while they return to their homes. Through this editorial, we prompt caution to the organizers and the event-hosting nation’s administration regarding the potential threat and suggest measures to avoid any medical emergencies related to COVID-19.

## Editorial

Mainland China hosted the World University Games 2023 under the banner of the International University Sports Federation (FISU) in Chengdu from July 28 to August 8, 2023. The Asian Games 2023 will ensue in the city of Hangzhou, China, at the Zhejiang University of Technology from September 23 to October 8, 2023. Due to the rapid spread of coronavirus disease-19 (COVID-19) in China, this 15-day-long mega continental event was rescheduled from being held in September 2022 to 2023. This Asian Games 2023 will feature an array of 61 disciplines and sports (482 events in all), where players from more than 25 participating countries will showcase their talents in their respective events. Big contingents representing most of the Asian countries are participating in various events. In light of this, the participating delegation including the contestants, as well as the sports enthusiasts and fans thronging from all around the world in large numbers, pose a great threat as “global spreaders” as and when the games wind up and the crowd disperses to the respective countries/locations. If not nipped in the bud, this could pose a critical community healthcare concern subsequently.

As per a recent report in the journal Nature, China shall witness an infection cycle every six months while the highly infectious variants are still around the corner and COVID-19 restrictions are lifted [1]. China witnessed a surge in SARS-CoV-2 infections in the early part of December 2022 after it relaxed its “zero-COVID” policy [2]. The rolling waves of infection pose a risk of the emergence of novel variants that could be potentially more infectious and rapidly transmitting. The latest surge is unlikely to topple the Chinese healthcare system, although there is a fear of hundreds of millions being infected. Although the massive surge of COVID-19 infections in China is feared to immediately spark the emergence of a “troubling” new variant, there is no such report of novel variants yet [3].

The Chinese people would expect to acquire natural active immunity or herd immunity in the coming months from the recent exposures. As SARS-COV-2 is observed to be rapidly evolving, such immunity could compel it to develop ways to evade immunoprotection. In view of this, genomic surveillance is crucial to tracking the possible next “variant of concern” (VOC), as many countries around the world have dismantled their surveillance efforts. The genome analyses of the COVID-19 samples collected locally in Beijing, China, between November and December 2022 reported the prevalence of two Omicron subvariants, BA.5.2 and BF.7, as responsible for more than 90% of the infections [2]. This might not reflect the status in other Chinese regions as all the collected samples were from Beijing only. Further, the analyses may not have detected variants that would have emerged after the policy change in China, as the case samples collected were between November 14 and December 20, 2022.

Currently, a fresh COVID-19 wave reportedly causing mild infection is reportedly spreading through the Asian continent (https://www.japantimes.co.jp/news/2023/04/14/asia-pacific/mild-covid-wave-across-asia/). Numerous Asian countries registered a rise in COVID-19 infections in recent times. The Asian wave is attributed to an XBB mix, a highly transmissible Omicron subvariant, according to the genome analyses of samples deposited to the Global Initiative on Sharing All Influenza Data (GISAID) in the first half of 2023. Although their transmission rate has been appreciably high, it is heartening that these subvariants have not yet caused widespread severe illness. Their low severity could also be due to the fact that most of the Asian population has acquired active immunity by now through prior infections or through vaccination drives.

The re-emergence of COVID-19 waves is expected in the “new normal” more so as the monitoring and surveillance pivot and the “COVID-appropriate behavior” curbs are dismantled. To address any health issue at the venue, during the recently completed University games, there were country-wise healthcare facilities at the event sites in China. Emergency cases were forwarded to the nearest hospital. An Indian athlete participating in the University Games had a fever on arrival and was provided with essential medical support after being quarantined. Fortunately, no SARS-COV-2 virus was detected, and he was declared fit to participate in the events thereafter.

The Southeast Asian region reported 18,000 new COVID-19 cases between June 5 and July 2, 2023, as per the recent available data (https://www.thehindu.com/sci-tech/health/southeast-asia-reports-a-69-reduction-in-covid-cases in-28-days-who-update/article67053394.ece), the highest number being reported from Thailand and Bangladesh. However, hospitalization and intensive care unit (ICU) admission cases decreased as the reported deaths during the period; higher deaths were reported from Thailand, Indonesia, and India.

The World Health Organization (WHO) currently tracks XBB.1.5 and XBB.1.16 as the two variants of interest (VOIs) and monitors the six variants BA.2.75, CH.1.1, XBB, XBB.1.9.1, XBB.1.9.2, and XBB.2.3 as variants under monitoring (VUMs) along with their descendent lineages. The subvariants could be severe on the non-vaccinated individuals, the old-age people, or the immunocompromised [4]. These novel sub-variants infecting even the vaccinated is concerning. On numerous fronts, from a possible less-effective vaccination/vaccine to the “zero-COVID” policy, tackling the COVID-19 pandemic the Chinese way seems a failure that possibly did not sufficiently allow herd immunity to be achieved organically. China currently has a high elderly population, many of whom did not receive the requisite booster dosage.

This Asian game/Asiad obviously shall attract a large crowd of sports enthusiasts and fans from all around, being an international event. China is undoubtedly a sports-loving nation. Thus, a massive crowd is expected at every event in each stadium, as has been the scenario during the recently concluded FISU games. Crowding could pose a real healthcare issue, especially in the backdrop of the seemingly never-ending COVID-19 pandemic. From an infectious disease perspective, mass gatherings could spread transmissible viruses and are a serious health concern that warrants attention.

The risk of getting infected with seasonal influenza (especially as the Asiad is being held in the months of September and October) and other infectious diseases like diarrhea, measles, hepatitis A and B, and multidrug-resistant (MDR) bacterial infections in a large floating population are highly expected [5-7], although no specific information regarding this, in particular, is reported yet.

It calls for meticulous healthcare planning, disease management, and seamless risk monitoring and assessment strategies to avoid untoward healthcare issues. Random screening and testing of international flyers and booster shots need critical attention. Strict surveillance for the early detection of new emerging variants that could possibly affect the local, regional, and international community is also crucial. Adverse health incidents around the venue could put significant pressure on the healthcare system with local and global ramifications. If strict health screening is not resorted to, sportspersons, fans, visitors, and the public in general may possibly import the disease to the venue from elsewhere.

However, separate medical support facilities are available at each playing venue, accommodation venue, and the village. In case of an emergency, a well-equipped hospital nearby is alerted to handle the players and the officials. Keeping surveillance of the city sewerage and the water supply is necessary for a possible early indication. Following COVID-appropriate behavior (like face-masking, hand-sanitizing, and avoiding unnecessary crowding) in and around the venue is recommended. Very high-level hygiene and sanitation were maintained at the recently concluded University Games.

The Chinese government and local health authorities, in collaboration with global health agencies like the WHO, may be required to remain extremely alert and must use electronic and social media to reach the global community to curtail any such untoward incident, thereby nipping the healthcare challenge at the bud.
